# Aroused with heart: Modulation of heartbeat evoked potential by arousal induction and its oscillatory correlates

**DOI:** 10.1038/srep15717

**Published:** 2015-10-27

**Authors:** Caroline Di Bernardi Luft, Joydeep Bhattacharya

**Affiliations:** 1 Goldsmiths, University of London, Department of Psychology, London, SE14 6NW, United Kingdom

## Abstract

Recent studies showed that the visceral information is constantly processed by the brain, thereby potentially influencing cognition. One index of such process is the heartbeat evoked potential (HEP), an ERP component related to the cortical processing of the heartbeat. The HEP is sensitive to a number of factors such as motivation, attention, pain, which are associated with higher levels of arousal. However, the role of arousal and its associated brain oscillations on the HEP has not been characterized, yet it could underlie the previous findings. Here we analysed the effects of high- (HA) and low-arousal (LA) induction on the HEP. Further, we investigated the brain oscillations and their role in the HEP in response to HA and LA inductions. As compared to LA, HA was associated with a higher HEP and lower alpha oscillations. Interestingly, individual differences in the HEP modulation by arousal induction were correlated with alpha oscillations. In particular, participants with higher alpha power during the arousal inductions showed a larger HEP in response to HA compared to LA. In summary, we demonstrated that arousal induction affects the cortical processing of heartbeats; and that the alpha oscillations may modulate this effect.

The idea that our internal bodily states drive our emotions, desires and thoughts is not new^1^, but the research into how the viscera and the brain communicate and to what extent visceral signals influence higher level cognition through the communication with cortical areas is still in its infancy. For instance, a recent study^2^ has demonstrated that the cortical responses to heartbeats before stimulus onset could influence the detection of a weak visual stimulus presented at near-threshold, thereby suggesting that the neural responses to heartbeat may be associated with the (visual) conscious experience^2^,^3^. This cortical response to the heartbeat can be investigated through an event-related-potential (ERP) component called *the heartbeat evoked potential* (HEP), which seems to represent the cortical processing of the heartbeat occurring about 200–600 ms (variable according to the study) after the R-peak of the ECG waveform^4^. The HEP is consistently correlated with interoceptive accuracy^4^,^5^,^6^,^7^,^8^,^9^, i.e., one’s ability to consciously count her/his own heartbeats^10^. The HEP increases by training cardiac awareness^11^. This possibility of HEP modulation by targeted interventions on cardiac awareness is quite relevant considering that the HEP amplitude is diminished in pathological conditions such as heart disease^12^, and depression^13^. The HEP is also sensitive to a range of psychological factors including motivation^14^, attention^15^, and pain^16^. Furthermore, the HEP is higher when the participants keep their eyes opened after cortisol infusion^17^. Taken altogether, it seems that situations or conditions with higher intrinsic levels of arousal are accompanied by a larger HEP. Here we consider arousal as a state of higher intensity of certain affective and cognitive states which can be associated with higher physiological activation (e.g. higher sympathetic nervous system reactivity)^18^. These two levels of arousal, physiological and cognitive, interact and can be mutually modulated. For instance, depressed subjects often present lower physiological arousal in response to stress^19^ or emotional arousal^20^, as they also show lower HEP^13^. Furthermore, the HEP was found to be higher during eyes-opened compared to the eyes-closed condition^17^, and when people were more motivated by external rewards^14^ or when they attend to their own cardiac signals^15^. The effects of arousal on the HEP can also explain the correlation between arousal level and interoceptive accuracy already observed in the literature^21^. Moreover, previous studies are consistent with the hypothesis that cardiac awareness is dependent on the arousal dimension of the emotional experience, not on its valence^22^,^23^,^24^.

However, the effect of arousal on the HEP has not been directly demonstrated so far. We addressed this issue by investigating the effects of arousal manipulation on the HEP by inducing high arousal (HA) and low arousal (LA) mood using a combination of music and pictures. In addition, we investigated whether individual differences in sensitivity to arousal mood induction, as measured by the HEP, are associated with other physiological markers such as heart rate variability and brain oscillations. After a previous study^25^ which found that participants with greater cardiac accuracy presented higher sympathetic reactivity to mental stress and higher subjective arousal during emotional picture viewing, we predicted that the participants with higher physiological responses (heart rate and oscillations) to the arousal mood induction will also present a more sensitive HEP in response to such manipulation.

## Results

**Figure 1:**
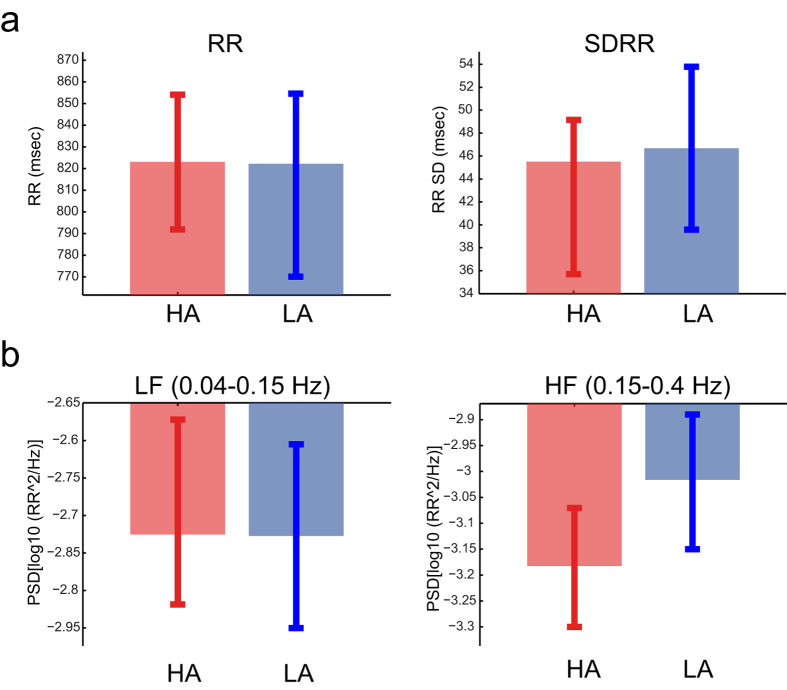
Heart rate variability during the high- (HA) and low-arousal (LA) conditions. The analysed HRV indexes are: (**a**) Interbeat-intervals (RR); (**b**) Variability of the interbeat-intervals (RR) as expressed by their standard deviations (SDRR); (**c**) Power spectral density (PSD) of the RR intervals in the low- (LF) and high-frequency (HF or RSA). The whiskers represent +/−1 SEM.

We monitored the EEG and ECG signals of adult healthy humans during two 5 minute sessions of HA and two 5 minute sessions of LA mood induction by using a combination of music and pictures (balanced for valence). In order to understand the effects of the arousal mood induction on the heart rate parameters, we extracted the interbeat intervals (RR) for each condition (HA vs. LA) and used it to estimate various HRV measures in both time (SDRR, RMSSD, PNN50) and frequency (LF, HF, and LF/HF) domains. We observed that the arousal mood induction was not associated with any change in the heart rate as estimated by the interbeat intervals (*t*_(15)_ = 0.22, *p* = .831) or in the time-domain HRV measures (SDRR: *t*_(15)_ = −1.16, *p* = .263; RMSSD: *t*_(15)_ = 0.59, *p* = .565; PNN50: *t*_(15)_ = 0.06, *p* = .950). However, in the frequency-domain, we found that the respiratory sinus arrhythmia (RSA – measured as the HF), an established marker of parasympathetic nervous system activation^26^,^27^, was significantly higher during the LA mood induction (*t*_(15)_ = 2.94, *p* = .010), but the other measures did not show any significant effect (LF: *t*_(15)_ = 0.86, *p* = .406, LF/HF: *t*_(15)_ = 2.09, *p* = .054). These results show that only the component related to the vagal activity (RSA) was sensitive to the arousal mood induction (Fig. 1).

**Figure 2:**
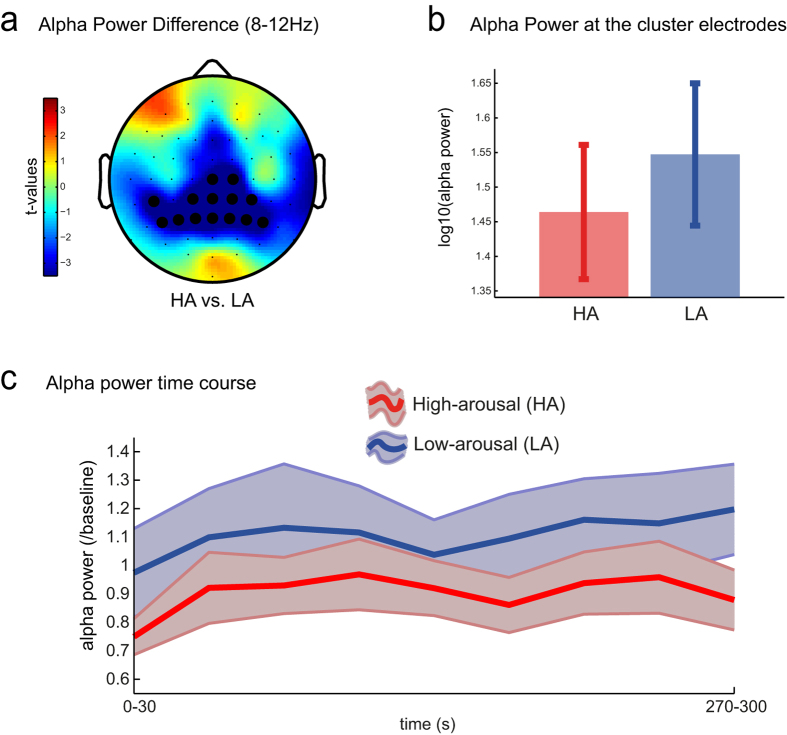
Alpha (8-12 Hz) power during high- (HA) and low-arousal (LA) mood inductions. (**a**) Topography of the differences in the alpha power; the significant cluster electrodes are highlighted in the figure (dark dots); (**b**) Error bars showing alpha power in the high- (HA: red) and low-arousal (LA: blue) conditions; (**c**) Time course of the alpha power (normalized by baseline (ratio) measured every 30 seconds (from 0 to 300 s). The shaded areas and the whiskers represent +/−1 SEM.

We then investigated the differences in ongoing brain activity during HA compared to LA mood induction. We estimated the power spectral density for each arousal condition in five classical EEG frequency bands (delta, theta, alpha, beta, and gamma, see *Methods*), and compared the two conditions by using non-parametric cluster permutation in order to reveal the significant clusters in each frequency band. We observed that alpha power was higher during the LA mood induction compared to the HA (alpha power averaged over the cluster electrodes: *t*_(15)_ = −5.98, *p* < .001) in the parietal electrodes (Fig. 2a,b shows the observed parietal cluster power differences). We estimated the time course of this effect by calculating alpha power over 10 non-overlapping time windows of 30 s each and calculated its change (ratio) in relation to the 2 s baseline period (i.e. the time period immediately before the onset of mood induction). The time course during the two conditions (Fig. 2c) shows that alpha power remained higher during LA than HA throughout the entire induction period.

**Figure 3:**
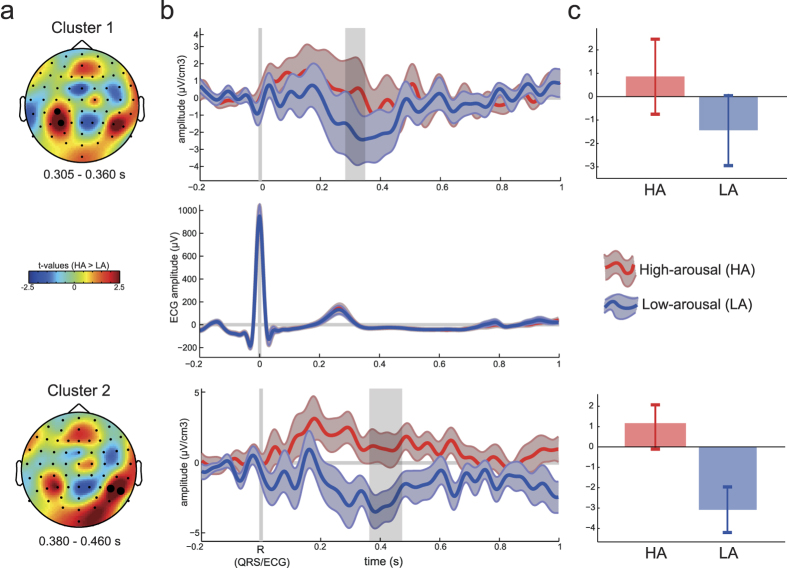
Heartbeat-evoked-potential (HEP) during high- and low-arousal mood induction. (**a**) Topography of the two clusters showing a significant difference in the HEP between HA (red) and LA (blue) mood inductions. The first cluster was found between 0.305 and 0.360 s after the R-peak of the ECG (top) and the second between 0.38 and 0.46 s. The highlighted electrodes remained significant during the whole cluster time-window. (**b**) The ERP waveforms in response to the heartbeats (R-peak) averaged over the cluster electrodes highlighted for the cluster (**A**) on the left-hand side during HA (red) and LA (blue) mood induction. The shaded areas represent +/−1 SEM, the area highlighted in grey is the time window in which the cluster remained significant. (**c**) Bar graphs showing the mean and +/−1 SEM of the ERPs averaged over the significant cluster electrodes (**a**) and time-window (**b**) for during HA (red) and LA (blue) mood induction. The top row of the figure represents the first cluster whereas the bottom represents the second cluster found.

Next, we investigated the impact of arousal manipulation on the HEP. Employing non-parametric cluster permutation test on HEP profiles revealed the presence of two clusters: an earlier one (Fig. 3a–c) over the left parietal from 200 to 300 ms after the heartbeat, and a later one (380–460 ms) on the opposite hemisphere (right parieto-temporal area). The earlier cluster (*p* = .041) was weaker but showed more positive amplitudes in response to the heartbeat in the HA condition (HA vs. LA contrast at the cluster electrodes: *t*_(15)_ = −3.50, *p* = .003). The later cluster (*p* = .008) was also higher for the HA compared to the LA (HA vs. LA contrast at the cluster electrodes P6 and P8: *t*_(15)_ = −3.96, *p* = .001) and it resembles the HEP (Fig. 3a–c, last row) in terms of waveform and time range (380–460 ms after the R-peak). There was no difference in the ECG waveform between the conditions (*t*-tests performed over each time point yield no significant results).

**Figure 4:**
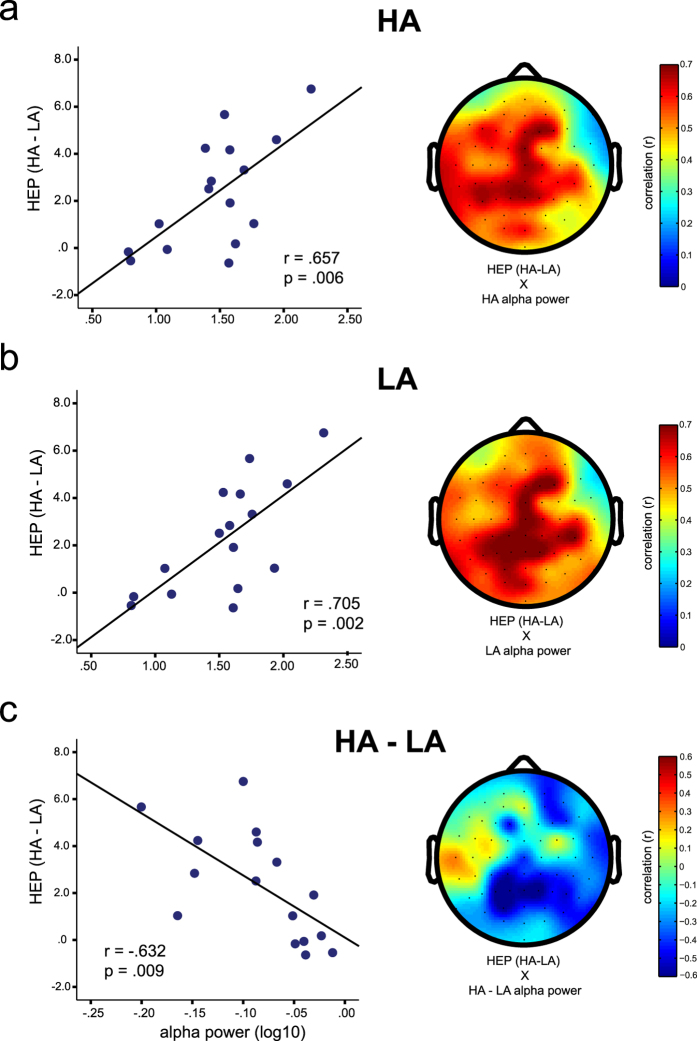
Correlations between alpha power and HEP difference between high- (HA) and low-arousal (LA) mood inductions. (**a**) Correlation between alpha power during HA and the HEP difference between HA and LA at the electrodes of cluster 2 (see Fig. 3); (**b**) The same correlation as in *a*, but for alpha power during LA mood induction; **(c)** Correlation between the alpha power difference between HA and LA and the HEP difference between these two conditions.

Finally, we explored the individual differences in the modulation of HEP by arousal in terms of the ongoing alpha oscillations. We tested the correlations between the alpha power during HA, LA and the difference between the two conditions (HA - LA) with the average HEPs over the electrodes and time window of cluster 2 (see Fig. 3) because it overlapped in time with the typical HEP profile observed in the previous studies^8^,^12^,^13^,^17^ and showed a more robust difference than the earlier cluster. We found that participants with higher alpha power also presented higher HEP sensitivity to the arousal mood induction (correlations with cluster 2 only, measured as the mean difference between HA and LA from 0.38 to −0.46 s after the R-peak in P6 and P8), in both HA (*r* = .657, *p* = .006) and LA (*r* = .705, *p* = .002) conditions (Fig. 4). In order to compare the correlations with HEP between the alpha power in HA and LA conditions, we used the Williams’ version of the Hotelling’s T test for comparing dependant correlations in small samples^28^ and observed a significant difference between the two correlations (*William’s t*_(13)_ = −2.19, *p* < .05), indicating a higher correlation between the HEP sensitivity and the alpha power during the LA induction. Interestingly, the HEP sensitivity to the arousal mood induction also correlated (*r* = .632, *p* = .009) with the alpha power difference between those two conditions (negative correlation as alpha was higher during LA). The higher alpha oscillations were modulated by arousal induction (lower during HA and higher during LA), the stronger the HEP was modulated during the same conditions. The topographs of these correlations (Fig. 4, right hand side) overlap with the topography of alpha power, indicating that the alpha power over the parietal and medial areas were correlated with how much the HEP changes in response to the arousal induction. Interestingly, there was no correlation between alpha power and the HEP amplitude during both HA (*r* = .321, *p* = .226) and LA (*r* = .070, *p* = .796), which indicates that the alpha oscillations may be behind how flexible the HEP is in response to arousal mood induction but not behind the amplitude of this ERP component per se.

### Control Analyses

To control for the possibility that the arousal induction effects on the HF and alpha oscillations resulted from the difference in the physical characteristics of the used stimuli, we compared these indexes between stimulation sets within each condition as there were two different sets for each stimulation (LA and HA sets A and B). We then compared the psychophysiological responses between sets A and B within each arousal stimulation category. If the physical characteristics of the stimuli alone are enough to affect these indexes, we would expect them to different across our stimulation sets. However, the results revealed no difference in the HRV (HF) between sets A and B for both high- (*t*_(15)_ = −1.29, *p* = .217) and low-arousal (*t*_(15)_ = 0.93, *p* = .368) inductions. In relation to the alpha oscillations, we also did not find any significant difference between the two sets for both HA (*t*_(15)_ = 1.15, *p* = .267) and LA (*t*_(15)_ = 0.70, *p* = .494).

In order to test whether the alpha effects were contingent to the heartbeats, we analysed the time frequency differences between HA and LA mood inductions. We compared the time frequency representations in response to the heartbeats by applying the non-parametric cluster permutation (HA vs. LA) to the time-frequency data. We used Morlet wavelets with 4 cycles on a time window from −1 to 1 s centred around the R-peak to estimate power from 4 to 45 Hz in steps of 1 Hz. The data were pre-processed in the same way as done with the ERP except that we did not apply low-pass filter and baseline correction. We estimated the power changes in each time and frequency point by using decibel transformation based on the pre- R-peak period (−0.2 to 0 s). We submitted the time-frequency representations from the R-peak until 1 s to a non-parametric cluster permutation using the same parameters as described in the *Methods*. We found no significant positive or negative clusters (*p* > .40), indicating that the differences observed in the alpha power during HA and LA are not contingent to the heartbeats. It is important to notice that the previously observed oscillatory difference between HA and LA conditions in this comparison was removed by normalizing the data by the baseline. Therefore, with this additional analysis we concluded that the oscillatory differences we found were probably unrelated to the heartbeat cycle but to the spontaneous fluctuations in such oscillations during the arousal induction.

Finally, it could be that the HEP differences between HA and LA were affected by the visual processing of low- and high-arousal IAPS pictures. In order to investigate this possibility, we repeated our analysis by removing the epochs whose heartbeats happened within one second following picture presentation. The results revealed the same effects for both clusters: on the first one from 200 to 300 ms after the heartbeat (HA vs. LA contrast at the cluster electrodes CP3 and P3: *t*_(15)_ = 2.77, *p* = .014), and on the second from 380 to 460 ms after the heartbeat (HA vs. LA contrast at the cluster electrodes P6 and P8: *t*_(15)_ = 3.49, *p* = .003). Therefore, these results support that our earlier effects were not related to lower level visual processing differences between the two arousal induction conditions.

## Discussion

In this study, we principally investigated the effects of arousal mood induction on the HEP recorded on the scalp and observed two major findings.

First, as predicted earlier, we found that HA was associated with larger HEPs and the effect was found over the right temporo-parietal electrode regions with most significant cluster over right parietal electrodes (P6 and P8). This difference was significant from 0.38 to 0.46 s after the R-peak, which is consistent with the time window observed in previous studies indicating the highest HEP amplitudes between 0.25 to 0.45 s after the R-peak^4^,^7^,^29^. The right temporo-parietal electrode region has been found to be modulated by emotional arousal^30^,^31^,^32^, and it has been suggested^33^ that this area plays a key role in modulating autonomic and behavioural aspects of emotion related arousal. Our results support this hypothesis and extend it to the autonomic domain showing that HA is associated with a higher response to heartbeats in the same electrode region, which might suggest that this area may be involved in the interface between the body and the brain correlates of arousal. However, this interpretation has to be taken with caution since this is an EEG study with limited spatial resolution and as such we cannot infer specific brain areas from the electrode space. Furthermore, a recent study^2^ investigated the MEG responses to heartbeats and found that a larger heartbeat cortical response at the right fronto-temporo-parietal regions was crucially associated with higher accuracy in detecting a fainted visual stimulus (grating), i.e. hit trials were associated with larger cortical response to the heartbeats in these areas. Source reconstruction of the heartbeat cortical field (MEG study) indicated that this effect was likely to be generated in the right ventral anterior cingulate cortex (vACC), medial prefrontal cortex (mPFC), and at the right inferior parietal lobe (right IPL). The right temporo-parietal electrode area was also found to be less responsive to emotional arousal in depressed subjects^20^, which might be one of the possible mediators for lower HEP found in depressed subjects compared to controls^13^. This result is also aligned with our earlier suggestion that arousal may be one of the key mediators of the various effects observed in the literature on HEP. For instance, motivation^14^, attention^15^, and cortisol during eyes opened condition^17^ are all associated with higher levels of arousal and also with higher HEP. In addition, arousal was found to be directly correlated with interoceptive accuracy^21^.

Second, we demonstrated for the first time, to our knowledge, a robust correlation between ongoing alpha oscillations and the modulation of the HEP by arousal. Briefly, we observed that participants with higher alpha power also showed a higher increase in the HEP in response to HA compared to LA condition, i.e. the higher the ongoing alpha power, the larger the HEP modulation by the arousal mood induction. Importantly, the observed correlation was across participants, and the alpha was shown to be correlated in both HA or LA, but higher during LA induction. This correlation might underlie previous findings that good heartbeat perceivers present higher sympathetic reactivity during mental stress and higher vagal reactivity during emotional picture viewing^25^. It might be that the ongoing alpha oscillations are important for the flexibility of the brain-body interactions. The topography of the correlations indicates that they were higher for parietal and medial alpha. Considering the well-established relation between cortical alpha rhythms and thalamic activity^34^,^35^, we suggest that participants with higher alpha have stronger thalamic activation which makes them more flexible or responsive to different mood induction by gating the visceral signals to the cortex. The thalamus might play a role in gating the visceral information coming from the nucleus tractus solitarii, a key area for visceral integration that receives inputs from the vagal nerve^36^. The thalamus is also an important gate for the sensory system and research in primates has shown that this area responded to vagus nerve stimulation^37^, supporting its role on processing afferent information from the heart. Further thalamus communicates visceral information to the insula, and medial prefrontal areas, including the genual and dorsal anterior cingulate cortex for a review^38^: A recent study^39^ showed that successful interoceptive learning (i.e. training related improvement in interoceptive accuracy) is mediated by the right insular cortex. Furthermore, they observed an increase in long-range gamma phase synchrony in participants who showed a correlated increase in objective interoceptive accuracy and metacognitive awareness after training. It is possible that the effects we observed over the parietal electrodes are associated to thalamus sending the information to anterior regions (insula and medial prefrontal cortex), which communicate with temporo-parietal areas. Future studies should target the connectivity between these brain areas in order to investigate whether arousal modulates the thalamic gating of visceral information to the parietal areas, or if these parietal areas receive the visceral information from anterior regions. Considering the inconsistency in the literature on the topography of the HEP^2^,^8^,^12^,^21^,^40^,^41^, it is also possible that the visceral information is communicated to various cortical areas which together form a percept of the heartbeat. In that model, every cortical area would be informed and process the cardiac information for different purposes. Note that although we suggest here that thalamus may play a role gating these visceral signals to other brain areas, our study do not allow us to test those claims due to limited spatial resolution of EEG. Nevertheless, the robust nature of the reported correlation between the alpha oscillations and the HEP component as modulated by the arousal induction indeed calls for future research to establish a causal link between these two brain responses.

It is important to stress here that our major findings cannot be explained by a mere difference in the heartbeat intervals between two arousal inductions. In fact, we did not find any significant difference in heart rate between the two conditions. Further, inducing HA vs LA did neither bring any change in other time-domain measures of heart-rate-variability (HRV, Fig. 1) nor in the ECG profiles (Fig. 3). However, the frequency domain index associated with vagal activity (HF) also known as the RSA, was significantly higher in the LA condition, consistent with the idea that lower levels of arousal are associated with higher vagal activity or vice-versa, i.e. higher levels of arousal associated with lower vagal activity. The RSA, or the high-frequency (0.15–0.4 Hz) component of the HRV, represents the synchrony between heart and respiration and it serves as an index of cardiac vagal activity (efferent vagal activity), controlled by the parasympathetic branch of the autonomic nervous system (PNS) for a review, see:^26^,^27^. A higher RSA during LA compared to HA is consistent with previous findings indicating that arousal, but not valence, is associated with the RSA^42^. In particular, the latter study found that lower arousal is associated with higher RSA, which suggests better vagal control in lower arousal situations. In this study, the conditions did not differ in terms of valence (as both positive and negative valence were equally present in the two conditions), which might explain why we did not find changes in the heart rate, as it seems to decrease in response to negative valence stimuli^43^.

Further to our HEP related findings, we also observed that LA was associated with higher alpha power over parietal electrode regions. This is in agreement with previous studies showing that higher-arousal is associated with lower alpha power^44^,^45^, which is expected considering that this oscillation is often inversely related to cortical excitation, especially in the parietal areas^46^,^47^. However, alpha oscillations are not only inversely correlated with the BOLD response in parieto-occipital areas, but also associated with increased thalamic activation^35^. In the present study, parietal alpha was higher during LA than HA mood induction, which could be associated with decreased cortical excitability and higher thalamic activation (specifically dorsal thalamus) since this brain area is deemed as one of the main sources of parietal and occipital alpha rhythms^34^,^35^. However, this generator is only hypothetical due to the low EEG spatial resolution. The alpha modulation by arousal mood induction seems to start early, already in the first 30 seconds of initial arousal stimulation, and importantly, it remains higher (LA compared to HA) during the whole induction period. The sustained increase in alpha oscillations could be the basis for more sustained changes in mood promoted by prolonged exposure to emotional stimuli as observed previously^48^. Altogether, our study shows that it is possible to modulate parietal alpha oscillations by inducing LA and HA combining music and pictures.

### Limitations

Considering the complexity of the brain-body interactions, we would like to mention some of the limitations of this study that can be further investigated in future studies. First, due to our focus on arousal, we combined both valences in the same protocol. Therefore, our results do not account for possible interactions with valence on modulating the heart-brain interactions. Second, we did not use self-report ratings of arousal. We did that in order not to interfere with the participants’ experienced emotions during the induction protocol as debriefing them after each run could cause them to focus more on those sensations in the following run. Therefore, our conclusions are limited to the effects of an arousal induction protocol which may have been perceived differently by each participant. Whether the effects of arousal induction on heartbeat evoked potentials are dependent on the subjectively perceived experienced arousal requires further investigation. Third, our difference in the HF parameter of the heart rate variability could have been caused by an increase in parasympathetic modulation of the heart during the LA, a decrease during the HA, or even a combination of both. Additionally, we did not control the respiration flow so we do not know whether these effects could be driven by changes in respiration rate during arousal mood induction. Finally, as an EEG study, we suffer from poor spatial resolution. A precise source for our HEP modulation in the cortex requires further investigation in future studies.

In summary, we demonstrated that heartbeat evoked potential as recorded on the scalp is modulated by arousal and the individual differences in the degree of HEP modulation are related with ongoing alpha oscillations. Additionally, we observed that these ongoing alpha oscillations are not time locked to the heartbeats, but spontaneous oscillations occurring during the arousal induction.

## Methods

### Participants

Nineteen healthy human volunteers (8 females; age range of 21–32 years) took part in this study. Two participants were discarded due to a failure in the equipment (data loss over 50%), and one due to high noise in the EEG reference electrodes. Data from the remaining 16 participants were analysed. This experiment was conducted in accordance with the ethical standards stated in the 1964 Declaration of Helsinki. All participants read and signed the informed consent before taking part in this study. The experimental protocol was approved by the local Ethics Committee of the Department of Psychology at Goldsmiths, University of London.

### Arousal Mood Induction Protocol

In order to induce HA and LA, we presented visual and auditory stimuli simultaneously because it has been shown that combining these two modalities leads to a much robust modulatory effects than either of the stimuli presented in isolation^49^. For the visual stimuli, 200 pictures were selected from the International Affective Picture System (IAPS) database^50^. One-hundred pictures with a mean arousal rating equal to or higher than 5.50 on a 9-point rating scale estimated using the Self-Assessment Manikin (SAM) rating system were used for the HA mood induction. The other 100 pictures with a mean arousal rating equal to or lower than 3.50 were used for the low-arousal mood induction^50^. The valence was counterbalanced across conditions (HA and LA), in order to have both positive and negative valenced pictures in the HA and LA mood inductions. For the auditory stimulation, 4 musical excerpts were used. The HA induction music excerpts were: Georges Bizet – Carmen Overture, and Mozart – Sonata for two pianos in D major K. 448. The low-arousal induction music excerpts were: Samuel Barber – Adagio for strings, op.11, and Albinoni – Adagio in G minor. These musical excerpts were previously validated as reliable mood inducers^51^,^52^,^53^. The duration of each mood induction was 5 min. Each musical excerpt was played while the respective pictures were being presented (in random order for 6 s each). Each participant took part in two inductions (5 minutes each) for each arousal condition (2 HAs and 2 LAs). The order of the condition was counterbalanced across participants (half of the participants started with HA and half with the LA induction) and alternated (a HA was always followed by a LA induction and vice-versa).

### EEG and ECG recording

EEG and ECG signals were acquired using a 64-channel (10–20 system) EEG system (ActiveTwo, BioSemi Inc.) placed in an electromagnetically shielded room. During the recording, the data was band-pass filtered between 0.16 and 100 Hz. The vertical and horizontal eye-movements (EOG) were monitored by electrodes above and below the right eye and from the outer canthi of both eyes, respectively. Additional external electrodes were placed on both left and right ear lobes as reference. The ECG was recorded using the two remaining external channels with a bipolar ECG lead II configuration. The sampling frequency was 512 Hz. The task was presented on a PC by using the MATLAB toolbox Cogent 2000 (http://www.vislab.ucl.ac.uk/cogent.php, Date of access: 11/02/2014).

### ECG data processing

The ECG data was processed using MATLAB based custom scripts for the analysis of heart rate variability (HRV) according to the recommended standards for HRV measurement^26^. The QRS complex was identified using a QRS detection algorithm based on filter banks which enables the identification of the complex by decomposing the ECG in sub-bands with uniform frequency bandwidths implemented in MATLAB with the code provided by the authors^54^. The ECG data was visually inspected to assure that the R-peaks were correctly detected. Using the R latencies, the inter-beat intervals (IBI) were obtained. These values were used to estimate the heart rate (RR) and the HRV indexes, which included both time-domain: the standard deviation of the RR (SDRR), the root mean square of successive RR intervals (RMSSD), the percent of normal to normal intervals with a difference higher than 50 ms (PNN50), and frequency-domain measures: low-frequency (LF: 0.04 to 0.15 Hz), high-frequency (HF: 0.15–0.4 Hz), and their ratio (LF/HF).

### EEG data processing

The EEG data were processed and analyzed using MATLAB custom scripts and the following toolboxes: EEGLAB^55^ for data preprocessing, including Independent Component Analysis (ICA), and Fieldtrip^56^ for the non-parametric cluster permutation analysis. The EEG signals were algebraically re-referenced to the arithmetic average of the right and left earlobes, high-pass filtered at 0.5 Hz. For each participant, four epochs (2 HA and 2 LA) of 302 seconds length (2 seconds before the onset of induction) were selected. Independent Component Analysis (ICA) was used to identify and remove the eye-blink artifacts. Subsequently, the data was visually inspected for other large artifact removal (e.g. muscle, saccades, etc.). Current source density (CSD) transformation (surface Laplacian) was subsequently applied to the data in order to attenuate the low-spatial frequency features from the data, especially the cardiac-field artifact (CFA), since it is spread across the scalp^57^. The CSD was applied using the CSD toolbox^58^ which computes the scalp surface Laplacian by applying the spherical spline algorithm^59^, in which the *G* (surface potentials) and *H* (current source densities) matrices are calculated based on a sphere. We used the default parameters including a smoothing parameter of 4 (*m* parameter) and a Legendre Polynomial of 10.

The power spectral density (PSD) was calculated using Welch’s method (averaged periodogram) by dividing the data into 2 seconds windows with an overlap of 50%; for each arousal condition, PSD was averaged across two inductions. We estimated the spectral power from 1 to 70 Hz with a step size of 0.5 Hz. The power values at each electrode for each condition were averaged over standard EEG frequency bands: delta (1–4 Hz), theta (4–8 Hz), alpha (8–12 Hz), beta (13–30 Hz), gamma (30–70 Hz), and subsequently log-transformed to normalize their distributions.

For the analysis of the HEP, the data was epoched around each identified R peak (200 ms before the R-peak to 1 s following it). Considering that the HEP is a very low frequency ERP component^4^, we low-passed filtered the data at 15 Hz. The CFA imposes a great challenge to the analysis of the HEP because the epoching and subsequent averaging the data on the R-peak of the ECG amplifies the CFA since it becomes time-locked to the heartbeat, especially during the QRS and the T-wave of the ECG^57^. In order to minimize the CFA, we used ICA and current-source density surface Laplacian transformation (described above). The ICA was used to correct for the CFA by removing the independent components (most often one, maximum two) whose timing and topography (occurring during QRS and T waves) over trials (similar across trials) resembled the characteristics of the CFA^13^.

### Statistical Analysis

Although we were mostly interested in the HEP, previous studies^2^,^8^,^12^,^21^,^40^ did not give robust features of the HEP which would allow to formulate clear hypothesis towards its topography as conflicting findings were found in its both time and spatial dimensions. Therefore, we adopted an exploratory approach, non-parametric cluster permutation^60^, to statistically compare the brain responses to HA and LA mood inductions; it is essentially a biologically inspired statistical approach for exploratory analysis of multidimensional neuroimaging data. This method has been successfully used in EEG studies^2^,^61^. Non-parametric cluster permutation is robust against false alarm or Type I error and more sensitive than other conservative solutions (e.g. Bonferroni’s correction) against Type II error. The principal idea behind the method is that if an effect is biologically relevant, it will be robust in a cluster on a multidimensional space (not necessarily highly significant at a single electrode/time point). In addition, the method tests the significance of the effect by comparing the actual statistical result for each comparison against a distribution of the statistical values obtained by comparing the same data points after shuffling or randomly allocating them to the two conditions. The latter procedure provides a good estimate of the actual chance levels, as the shuffling is based on the same data (i.e. if there is a bias on the chance level, it will subsequently show up in the distribution of the shuffled data).

In order to find the clusters, the following steps are taken: 1) *t*-statistics (in this case between HA vs. LA) for each of the samples in the multidimensional data structure; 2) threshold these sample-specific statistics by its *p*-value (*p* < .05); 3) find neighbouring data points that (i) exceed the threshold and (ii) have the same sign; 4) calculate the cluster-level statistics by taking the sum of the *t*-statistics; 5) take the maximum of the cluster-level statistics; 6) evaluate this maximum under its permutation distribution. We used this technique to compare the HEP waveforms between HA and LA conditions. The permutation distribution was derived from the statistic values of dependent *t*-tests, based on 500 random permutations. The threshold for the inclusion in the cluster was set at 0.05. For the comparisons involving ERPs, the cluster permutation (large versus small errors) was run considering the spatial (electrodes) and temporal dimensions (time-points). For the comparisons involving TFRs (control analysis described at the end of the results section), the clusters were calculated based on the spatial, temporal and spectral (frequencies) dimensions. We considered electrodes with a distance of less than 5 cm as neighbours (yielding on average 4.2 neighbours per electrode).

## Additional Information

**How to cite this article**: Luft, C. D. B. and Bhattacharya, J. Aroused with heart: Modulation of heartbeat evoked potential by arousal induction and its oscillatory correlates. *Sci. Rep*. **5**, 15717; doi: 10.1038/srep15717 (2015).
